# Comparing Explicit and Implicit Measures for Assessing Cross-Cultural Food Experience

**DOI:** 10.3389/fnrgo.2021.646280

**Published:** 2021-03-22

**Authors:** Daisuke Kaneko, Ivo Stuldreher, Anne J. C. Reuten, Alexander Toet, Jan B. F. van Erp, Anne-Marie Brouwer

**Affiliations:** ^1^Kikkoman Europe R&D Laboratory B.V., Wageningen, Netherlands; ^2^Microbiology and Systems Biology, Netherlands Organisation for Applied Scientific Research TNO, Zeist, Netherlands; ^3^Perceptual and Cognitive Systems, Netherlands Organisation for Applied Scientific Research TNO, Soesterberg, Netherlands; ^4^Human Media Interaction Lab, University of Twente, Enschede, Netherlands

**Keywords:** cross-cultural, response bias, explicit, implicit, physiological measures, HR, EDA, sip size

## Abstract

The present study investigated the potential of implicit physiological measures to provide objective measures of affective food experience in contrast to explicit self-report ratings in a cross-cultural context. Dutch and Thai participants viewed 120 food images portraying universal food image categories (regular and molded food) and cultural food image categories (typically Dutch and Thai food). The universal food images were taken as ground truth high and low valence stimuli, where we assumed no genuine difference in affective experience between nationalities. In contrast, for the cultural food images, we did expect a genuine difference between nationalities. Participants were asked to rate valence, arousal and liking of each food image. In addition, heart rate (HR) and phasic electrodermal activity (EDA) responses to the images were recorded. Typically Asian and Western response biases were found for explicit ratings of regular and molded food with an extreme response style for Dutch, and a middle response style for Thai participants. However, such bias was not observed in HR. For cultural food image categories, HR showed the hypothesized interaction between participant nationality and food image category, reflecting the expected genuine difference between nationalities in affective food experience. Besides presenting participants with images, we also asked participants to taste typically Thai and Dutch drinks. Similar to images, a significant interaction between participant nationality and cultural food category was found for HR. An interaction was also found for sip size, while this was not seen in explicit measures. We attribute this to differences in the moment that these measures were taken. In this study, phasic EDA did not appear to be a sensitive measure of affective food experience, possibly since stimuli mostly differed in valence rather than arousal. To conclude, our study constitutes an example where cultural bias negatively affected the accuracy of self-reports, and only the implicit physiological measures followed the prior expectations of genuine food experience, indicating the potential of these measures to study cross-cultural food experience.

## Introduction

To predict whether consumers will choose a certain food product, their emotional response when experiencing this product is considered to be an important predictor (Dalenberg et al., 2014; Gutjar et al., 2015; Köster and Mojet, 2015; Samant et al., 2017). With the current globalizing trend, measuring these emotions cross-culturally is important for international food marketers (Rozin, 1988, 2006; Meiselman, 2013, 2015).

Assessment of food-evoked emotions is predominantly based on explicit measures (“conscious” self-report ratings) rather than on implicit measures (“unconscious” physiological and behavioral measures) (Lagast et al., 2017; Kaneko et al., 2018). Explicit measures are relatively easy to apply, practical for quantitative analysis, and widely believed reliable for most sensory and psychological studies (Lawless and Heymann, 2010; Dorado et al., 2016). Many large international companies explore food product-elicited emotions across different countries by simply translating emotion questionnaires into multiple languages (Meiselman, 2015). However, the drawback of using explicit self-report questionnaires for assessing affective experience cross-culturally is that cultural background influences how people “self-report,” what emotional language they use, and how they use rating scales to describe their own food-evoked emotions (Meiselman, 2015; Van Zyl and Meiselman, 2015, 2016; Silva et al., 2016; Ares, 2018). A review by Meiselman (2015) questioned whether simple translations between languages capture the local meaning of emotional words in a questionnaire well enough – people raised in different cultures may not experience the same emotions evoked by the same stimuli, and evoked emotions may not be expressed in the same manner. For example, Uchida and Kitayama (2009) analyzed American and Japanese descriptions of “happiness” and showed that Japanese associate happiness with “social harmony” while Americans associate it with positive experience of personal achievement. Concerning culture-dependent use of rating scales, Western respondents have been found to have an “Extreme Response Style” (ERS) (using the extremes of rating scales), whereas Asian respondents more often use a “Middle Response Style” (MRS) (using the neutral part of the scale) (Chen et al., 1995; Harzing, 2006; Kaneko et al., 2019b). Problems with translating between languages, and intercultural differences in terms of using rating scales, could potentially be overcome by implicit measures, which reflect fast, non-conscious, and uncontrollable responses (Soto et al., 2005; Lagast et al., 2017; Ares, 2018; Kaneko et al., 2018).

Several physiological measures have been studied in the context of probing affective experience when tasting and viewing food or food images. Among them, heart rate (HR) and electrodermal activity (EDA) are the most often used implicit physiological measures in recent consumer research. These measures have been shown to distinguish between tasting different beverages, chocolates, liked, and disliked food (de Wijk et al., 2012; Danner et al., 2014; Torrico et al., 2018; Kaneko et al., 2019a). Outside the food domain, several studies have compared implicit physiological responses to different types of stimuli between individuals from different cultures. One study showed weaker electrodermal responses to disgust-eliciting film clips in Asian-American compared to European-American participants (Soto et al., 2016). No difference between cultural groups was found for physiological responses to stimuli such as acoustic startle [Chinese-American and Mexican-American groups; (Soto et al., 2005)], emotional films [Chinese-American and European-American groups; (Tsai et al., 2000)], and reliving of intense emotional episodes [Hmong-American and European-American groups; (Tsai et al., 2002)]. These results may be taken to mean that Asian-Americans and other Americans “really” differed in emotional experience when watching disgust-eliciting movies, and not when experiencing the other types of stimuli.

To the best of our knowledge, only two studies in the food domain used implicit measures to investigate psychophysiological effects of food products between different nationalities [Asian who spent <2 years in Australia and Australian groups; (Torrico et al., 2018, 2019)]. These studies used rating scales as well as a camera to monitor heart rate, skin temperature and facial expressions to investigate cross-cultural effects of viewing universal and culture-specific food (Torrico et al., 2018, 2019). Their results indicated that rated food liking is positively correlated to familiarity, and that skin temperature differentiates between cultural groups when tasting culture-specific food samples (Torrico et al., 2019) while no physiological differences between cultural groups exist when tasting (universal) chocolate samples (Torrico et al., 2018). These results are in line with the idea that physiological measures reflect the “true” emotion: cultural groups do not differ when tasting universal stimuli, and they do differ (in skin temperature) when tasting samples that are expected to genuinely elicit different emotions. No physiological effects were found besides skin temperature, but we should note that camera-based analysis is usually less precise and suffers more from artifacts caused by (chewing) movements, head orientation, and lighting conditions compared to traditional sensors (Kranjec et al., 2014; Bach et al., 2015; Hassan et al., 2017).

In the present study we investigated whether implicit physiological measures (HR and phasic EDA recorded using traditional sensors) can contribute to comparing affective food experiences across cultures objectively without cultural response biases that affect explicit self-report methods. We compared explicit and implicit responses between two cultural participant groups, Dutch (representative for ERS) and Thai (representative for MRS), toward universal food images (regular and molded food) and cultural food images (typically Dutch and Thai food). We selected universal and cultural food image categories so that we could assume a genuine difference in emotional experience between Dutch and Thai participants for the two types of cultural foods, but no genuine difference for the two types of universal foods. For the latter category, we can safely assume a ground truth affective experience of low valence (unpleasant) and high arousal for the molded compared to the regular food images, in both Dutch and Thai participants. A lack of effect of universal food category would therefore indicate that the measure is insensitive. No differences between the two nationalities are expected for implicit measures for universal food image categories while we expect differences on explicit measures due to culturally-dependent response bias. On the other hand, we expect response differences between Dutch and Thai participants on both explicit and implicit measures for the cultural food image categories. Viewing food images happens in real life, and is practical in (psychophysiological) experiments in which a large number of trials is desirable. However, viewing food images is expected to elicit different responses than being confronted and tasting real food (de Wijk et al., 2012). To extend our study beyond viewing food images, we also examined implicit and explicit affective responses in Dutch and Thai participants when tasting typically Thai and typically Dutch drinks.

Our specific hypotheses are as follows:

1) Nationality affects explicit measures for universal food images (regular and molded food) due to a culturally dependent response bias. ERS is expected for Dutch participants, and MRS for Thai participants.
2) Nationality affects explicit measures for the cultural food images due to a genuine difference in affective experience caused by a difference in familiarity with the types of food (in addition to a culturally dependent response bias), resulting in an interaction between participant nationality and cultural food category.
3) Implicit physiological measures are affected by universal food image category (regular vs. molded food), but the effect is the same for both nationalities (i.e., no interaction between participant nationality and universal food image category).
4) Implicit physiological measures toward cultural food image categories (Dutch and Thai food) reflect genuine differences in affective experience between participant groups, resulting in an interaction between participant nationality and cultural food image category.
5) Similar to food images, explicit and implicit responses to tasting cultural drinks show an interaction effect between participant nationality and cultural drink category.

## Methods

### Participants

42 Thai participants were recruited from Chulalongkorn University in Thailand and 45 Dutch participants were recruited from the participant pool of the research institute where the main part of the research was conducted (TNO Soesterberg, The Netherlands). The recruitment process excluded people with color vision deficiencies; food allergies; diets such as vegetarian, vegan or religion-related; an immigration background; an eating disorder diagnosed in the last 3 years. Also, people who had visited The Netherlands (for Thai participants) or Thailand (for Dutch participants) and who had lived abroad for more than 1 month could not participate. Participants were asked not to eat for 1 h before testing. The experimental protocol was approved by the TNO Institutional Review Board (Ethical Approval Ref: 2019-033) and was in accordance with the revised Helsinki Declaration (World Medical Association, 2013). All participants signed an informed consent sheet before the experiment started and received a reward to thank them for participating in the study after completing the experiment.

### Materials

#### Food Images

**Figure 1:**
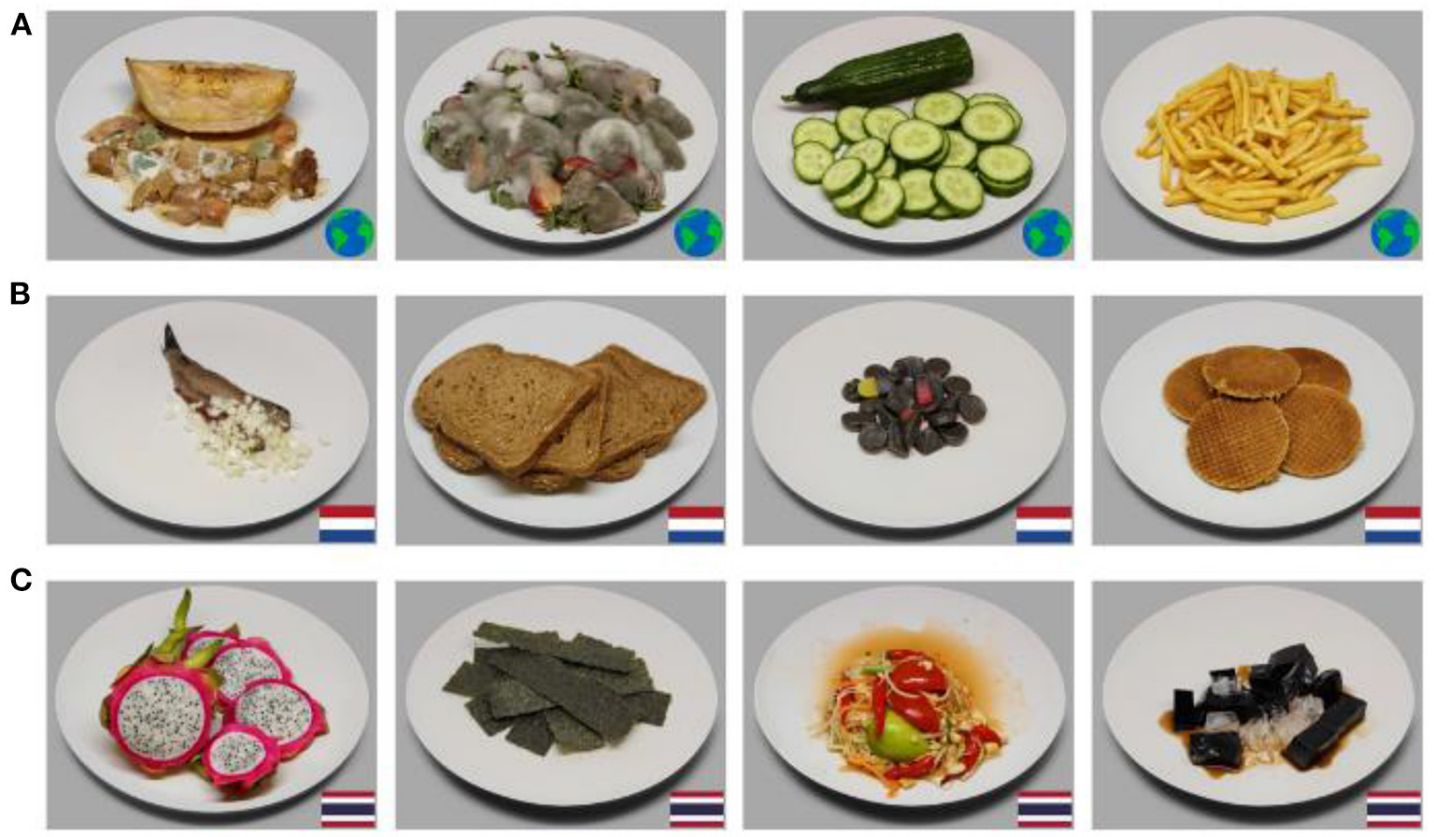
Stimulus examples of **(A)** universal molded food images (melon and strawberries) and universal regular food images (cucumber and French fries), **(B)** Dutch food images (herring, wheat bread, liquorice candy, stroopwafels), and **(C)** Thai food images (dragon fruit, seaweed chips, som tam, grass jelly).

Food images were selected from the Cross Cultural Food Image Database (CROCUFID) (Toet et al., 2019), which is a collection of food and non-food images, photographed on a standardized plate using a standardized photographing protocol (Charbonnier et al., 2016). From this database, we selected 60 “universal” food images which are expected to be familiar to participant of both nationalities (47 regular food images and 13 molded images); and 60 “cultural” food images (30 typically Dutch and 30 typically Thai food images). As shown in Figure 1, the national flag of the food's origin was presented on the right bottom of each image to ensure participants recognizing and interpreting the food in a similar way. Universal dishes were accompanied by an image of a globe.

#### Drinks

As a typically Dutch drink, a popular long seller yogurt drink, Fristi (Friesland Campina B.V. the Netherlands), was used. As a typically Thai drink we selected a Chrysanthemum tea drink (Vitasoy International Holdings Limited, Thailand). Both drinks were served in white plain cups.

#### Rating Scales and Implicit Behavioral Measure

The following rating scales were used to rate emotions evoked by viewing food images and tasting drinks, and to check for the familiarity of the participants with the stimuli:

##### EmojiGrid

**Figure 2:**
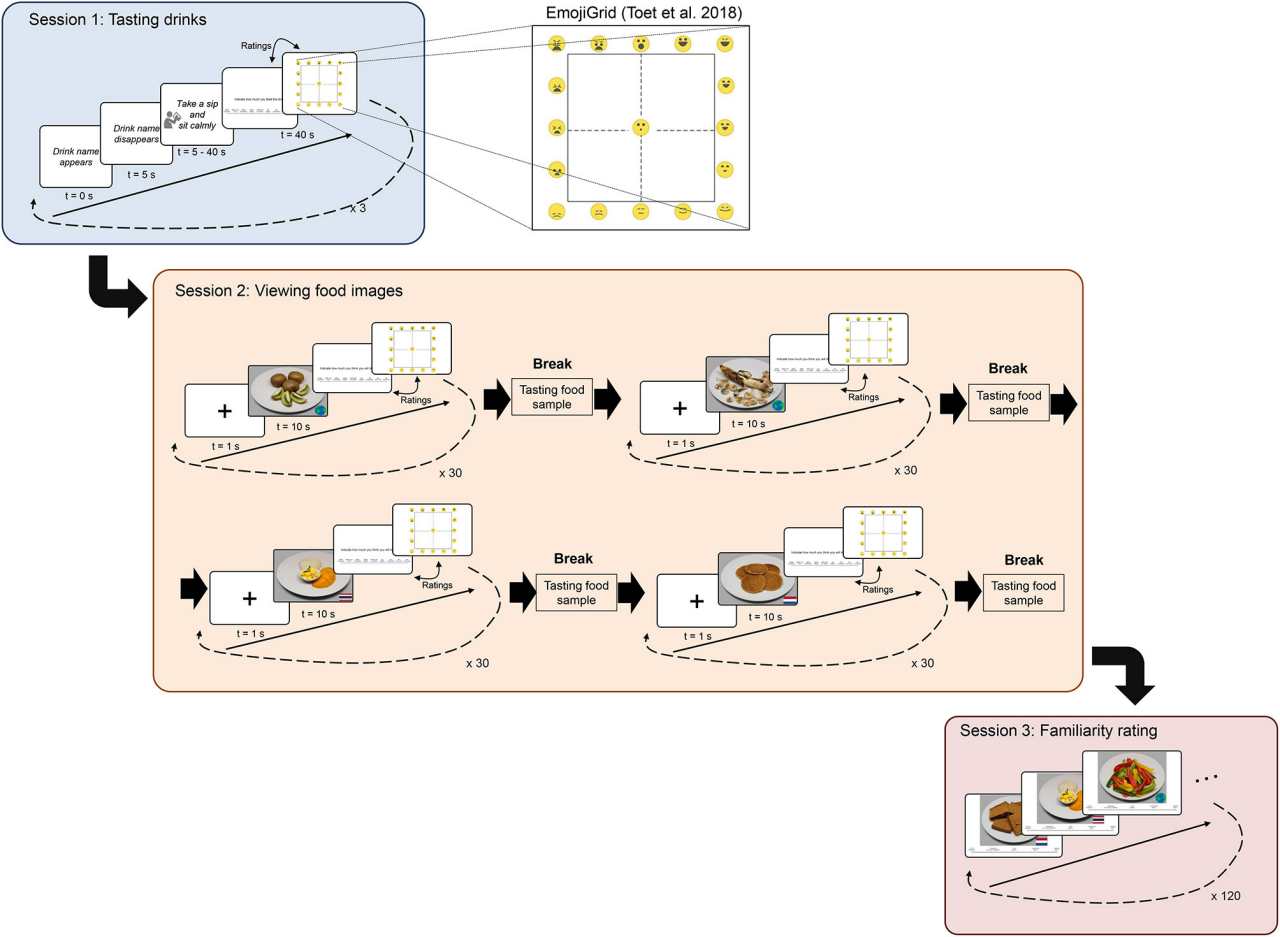
Schematic overview of an experimental trial and of the experimental procedure. All participants followed the exact same procedure. Physiological response data were collected during session 1 and 2. At the center, the EmojiGrid (Toet et al., 2018), used for rating valence and arousal is depicted.

An intuitive visual self-report tool that has been specifically developed for the assessment of food-evoked emotions and that has shown to be suitable for cross-cultural testing (Toet et al., 2018; Kaneko et al., 2019b). Participants report their emotion by clicking the appropriate location in the grid, where each location is associated with a valence and arousal score, ranging from 0 (lowest) to 100 (highest). The Emojigrid is depicted in Figure 2.

##### Hedonic Liking Scale

9-point scale with anchors for each point. The anchors are: (1) “dislike extremely,” (2) “dislike very much,” (3) “dislike moderately,” (4) “dislike slightly,” (5) “neither like nor dislike,” (6) “like slightly,” (7) “like moderately,” (8) “like very much,” and (9) “like extremely” (Lim, 2011).

##### Familiarity Scale

5-point scale with anchors for each point. Anchors of this five-point scale were labeled: (1) “I do not recognize it,” (2) “I recognize it, but I have not tasted it,” (3) “I have tasted it,” (4) “I occasionally eat it,” (5) “I regularly eat it” [adapted from Tuorila et al. (2001)]. This scale was used to check whether Thai and Dutch cultural food images were more familiar to participants of the matching nationality compared to the other nationality.

A scale was used during the tasting session to measure sip size of both cultural drinks by weighing the drink before and after the participant had taken a sip, following the procedure in a previous study (Kaneko et al., 2019a).

#### Physiological Recording Equipment (Electrocardiogram and Electrodermal Activity)

Electrocardiogram [ECG; for heart rate (HR)] and electrodermal activity (EDA; for phasic EDA) were recorded using an Active Two MkII system (Biosemi B.V., Amsterdam, the Netherlands), with a sampling frequency of 512 Hz. ECG electrodes were placed on the right clavicle and on the lowest floating left rib. EDA was measured by placing gelled electrodes on the fingertips of the index finger and the middle finger of the non-dominant hand. Two reference electrodes were attached to the temporal bone behind the ears.

### Experimental Design and Procedure

After participants arrived at the laboratory (depending on the nationality, either located at Chulalongkorn University, Bangkok, Thailand; or TNO Soesterberg, the Netherlands), they were told that the experiment consisted of a “tasting session,” a “viewing session,” and a “rating familiarity session.” The experimenter also explained that ECG and EDA sensors would be attached to measure HR and EDA during the experiment. Participants signed the informed consent form and were seated in a comfortable chair in front of an experimental presentation notebook. Then, the ECG, EDA, and reference electrodes were attached, and all signals were checked. HR and EDA were recorded during the tasting and viewing sessions. A schematic image of the study is shown in Figure 2. The total duration of the experiment was ~75 min.

#### Tasting Session

The procedure of the tasting session followed that used in a previous study (Kaneko et al., 2019a). Before the tasting session started, the experimenter showed and explained how to take a sip and to put the cup down after the sip, and participants performed a practice trial with water. After this there was time for additional practice or instructions when needed. The testing procedure started with the presentation of the name of the drink on the screen. This was the sign for the experimenter to place the appropriate drink in front of the participant. After 5 s, the name of the drink disappeared, which was the sign for the participant to take one sip. After taking the sip, the participant put the cup down, sat still and looked at a blank white screen. Thirty-five seconds after the name of the drink had disappeared from the screen, the EmojiGrid and hedonic scale appeared in successive, randomized order. Order was randomized so that the ratings from the two scales could be compared and would not be confounded by a possible order effect. After rating, the name of the next drink appeared on the screen. This procedure was repeated until three drinks had been served: first water for practice, followed by the Thai and Dutch drinks in counterbalanced order.

#### Viewing Session

After a short break, the instruction for the viewing session appeared on a screen, and participants had a chance to ask any questions to the experimenter. In this session participants viewed a total of 120 food images in two counterbalanced blocks (universal and cultural food image categories) each consisting of 60 randomized images. A fixation cross was presented for 1 s, after which a food image was presented for 10 s. After 10 s of viewing time, the EmojiGrid and hedonic scale appeared in successive, randomized order. Participants had unlimited time to provide their ratings using the mouse but were instructed to follow their initial impression. In an effort to increase participants' engagement with the images, they were told before starting the viewing session that they would be asked to taste some of the depicted food images they rated during each of four breaks. This part of the instruction was formulated in such a way that the participants were led to believe that they could be asked to eat an unpleasant (molded) food item: “There are four short breaks in this session. During these breaks, we will serve you one of the foods depicted in the images that you just saw. We ask you to taste this food. You are permitted to refuse, but we hope you will taste it.” Tasting breaks were introduced after each half block (30 images), and tasting samples were sliced banana pieces, peanut chocolate candies, a seaweed chip (typically Thai food item) and a small “stroopwafel” (typically Dutch food item).

#### Familiarity Rating Session

After finishing the viewing session, participants were instructed to rate their familiarity with all drinks and all food images they rated in the previous sessions. For drinks, the name of the drinks appeared successively on the screen in random order, accompanied by the familiarity scale. Actual tasting was omitted. Food images also appeared successively in randomized order, accompanied by the familiarity scale. Lastly, participants filled out a short demographic questionnaire, asking about age, gender, height, and weight.

### Physiological Data Processing

Data from three Thai participants were discarded due to failure of physiological recordings. Data processing was done using Matlab 2020a software (Mathworks, Natick, MA, USA).

Inter-beat intervals were extracted from ECG following Pan and Tompkins (1985) using a Matlab implementation from Sedghamiz (2014). By inversing the inter-beat interval, a heart rate semi-time series was obtained. Intervals exceeding the absolute threshold of 160 bpm or intervals deviating more than 20% from the previous interval were removed. The heart rate semi-time series was transformed to a regular timeseries at a 10 Hz sampling frequency using a piecewise cubic spline interpolation.

Raw EDA was down-sampled to 10 Hz. Continuous Decomposition Analysis as implemented in the Ledalab toolbox for Matlab was used to separate the tonic (slow) and phasic (fast) components of the EDA (Benedek and Kaernbach, 2010). In further analysis, only the phasic component is considered.

For food images, the pre-processed continuous physiological data were divided in epochs that were time-locked to stimulus onset for each image and each participant. The epoch comprises data ranging from fixation-cross onset to 10 s after fixation-cross offset. Response traces were baseline corrected based on the average value of the 1 s that the fixation-cross was presented.

For drinks, the pre-processed continuous physiological data were divided in epochs ranging from 5 s after drink name onset to 35 s after drink name offset (which was the sign for the participant to pick up the cup and take a sip), for each drink and each participant. Response traces were baseline corrected based on the average value of the 5 s during drink name onset.

Outliers were detected using Matlab's “isoutlier” function. Image epochs for which the average physiological response value was more than five median absolute deviations away from the median value across images were removed. This resulted in a removal of 0.06% of the HR data and 8.6% of the phasic EDA data. No outlier detection was conducted for physiological response value for drinks.

Grand-average response traces were obtained for each participant and each of the food image categories [universal food image category (regular and molded), cultural food image category (Dutch and Thai), and cultural drink category (Dutch and Thai)] by averaging over all data traces corresponding to the food image categories of each participant. We then computed the mean HR and mean phasic EDA for each participant and each food image category over the 10 s following image onset and over 35 s following drink name offset.

### Statistical Analysis

Statistical analysis was conducted with SPSS ver. 25 (IBM, USA). Two-way mixed ANOVAs were performed on each explicit and implicit measure in response to the universal food images, the cultural food images and the cultural drinks. In all of these analyses, the between-subject factor was participant nationality (Dutch or Thai), and the within-subject factor was respectively universal food image category (regular and molded); cultural food image category (Dutch and Thai); and cultural drink category (Dutch and Thai).

## Results

### Demographics

Table 1 shows the demographic descriptives for Dutch and Thai participants. The two groups did not differ in age [*t*_(85)_ = 1.662, *p* = 0.100] and BMI [*t*_(85)_ = 1.022, *p* = 0.310] according to Welch's *t*-test.

**Table 1:** Demographic variables for the Dutch and Thai participants.

**Cultural group**	***N***	**Female**	**Male**	**Age**	**BMI**
Dutch participants	45	28	17	21.2 (±1.7)	22.29 (±2.77)
Thai participants	42	24	18	20.6 (±1.5)	21.58 (±3.60)

### Familiarity Scores

**Figure 3:**
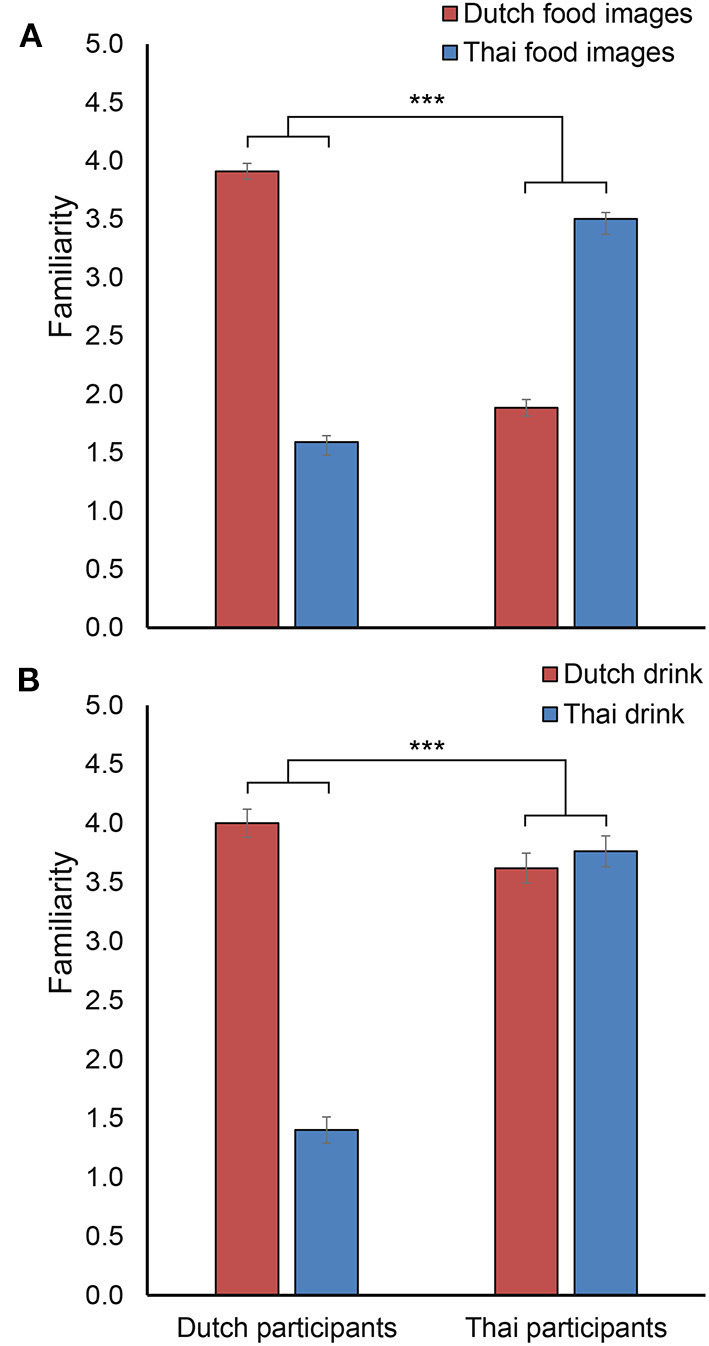
Mean familiarity scores of **(A)** Dutch and Thai food images and of **(B)** Dutch and Thai drinks rated by Dutch and Thai participants. Error bars indicate standard error of the mean. ^***^ indicates a significant interaction between participant nationality and cultural food category with *p* < 0.001.

Familiarity ratings are presented in Figures 3A,B.

Figure 3A shows the expected pattern of Dutch participants rating Dutch food as more familiar than Thai food and Thai participants rating Thai food as more familiar than Dutch food. This pattern is statistically corroborated by a significant interaction between participant nationality and cultural food image category [*F*_(1, 85)_ = 1299.52, *p* < 0.001, $\eta_{\text{p}}^{2}$ = 0.939], and indicates that the selection of cultural food images was appropriate. The two-way mixed ANOVA showed no significant main effect of participant nationality on familiarity scores [*F*_(1, 85)_ = 0.69, *p* = 0.407], and a significant main effect of cultural food image category [*F*_(1, 85)_ = 41.19, *p* < 0.001, $\eta_{\text{p}}^{2}$ = 0.326] on familiarity scores, with Dutch food images receiving higher familiarity ratings overall. As a comparison, regular food images from the set of universal food images, received very similar familiarity ratings as food images from the own culture: they received an average score of 4.1 from Dutch participant and an average score of 3.4 from Thai participants.

Figure 3B and the statistical analysis showed the expected interaction between participant nationality and cultural drink category [*F*_(1, 85)_ = 147.88, *p* < 0.001, $\eta_{\text{p}}^{2}$ = 0.635] even though familiarity scores for the Dutch and Thai drinks were similar for Thai participants, indicating that Thai participants reported to be quite familiar with yogurt drinks. The analysis showed a significant main effect of cultural drink category [*F*_(1, 85)_ = 118.68, *p* < 0.001, $\eta_{\text{p}}^{2}$ = 0.583] on familiarity scores, where the Dutch drink received overall higher familiarity ratings than the Thai drink. In addition, there was a significant main effect of participant nationality [*F*_(1, 85)_ = 57.19, *p* < 0.001, $\eta_{\text{p}}^{2}$ = 0.402], with Thai participants rating significantly higher familiarity scores than Dutch participants.

### Explicit Measures for Universal and Cultural Food Categories (Hypotheses 1 and 2)

#### Universal Food Category

**Figure 4:**
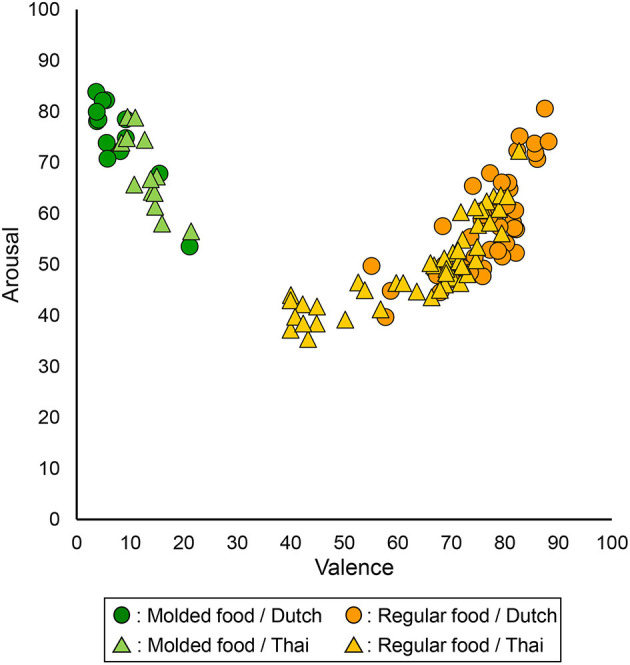
Mean valence (x-axis) and arousal (y-axis) scores for all 60 universal (regular and molded) food images. Each data point represents the mean score from either Dutch or Thai participants for each food image.

The mean Emojigrid valence and arousal scores for all universal food images (regular and molded) rated by both Dutch and Thai participants are plotted in Figure 4. The figure shows the typically found *U*-shaped distribution between valence and arousal scores (Toet et al., 2018; Kaneko et al., 2019b). As expected, scores for molded food images are situated on the left (low valence) while scores for regular food images are on the right (high valence). The expected culture-dependent response bias was observed in this figure: Thai participants use a smaller portion of the valence and arousal scale compared to Dutch participants. Dutch rated molded food images as more extreme in low valence, and regular food images more extreme in high valence than Thai participants; and Dutch participants rated both images as more arousing compared to Thai participants. These observations are confirmed by the following figures and analyses.

**Figure 5:**
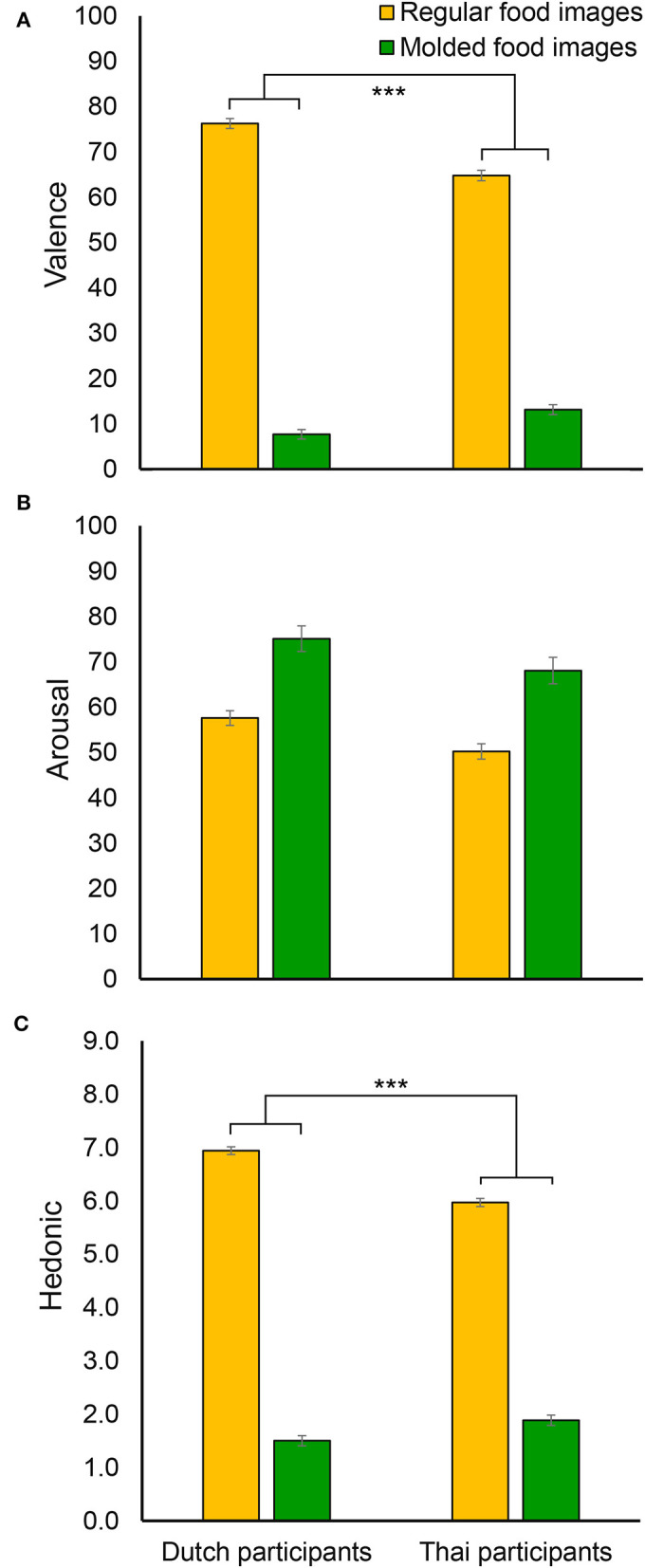
The averaged valence **(A)**, arousal **(B)**, and hedonic **(C)** scores for each participant nationality (Dutch and Thai) and universal food image category (regular and molded). Error bars indicate standard error of the mean. ^***^ indicates a significant interaction between participant nationality and universal food image category with *p* < 0.001.

Figures 5A,B represent average valence and arousal scores for each participant nationality and universal food image category. The two-way mixed ANOVA on valence scores showed a significant interaction between participant nationality and universal food image category [*F*_(1, 85)_ = 51.32, *p* < 0.001, $\eta_{\text{p}}^{2}$ = 0.376], indicating that Dutch participants rated valence more extremely (relatively higher valence rating for regular food images and relatively lower valence rating for molded food images compared to Thai participants). There was also a significant main effect of food image category [*F*_(1, 85)_ = 2583.37, *p* < 0.001, $\eta_{\text{p}}^{2}$ = 0.968], indicating lower rated valence for molded than for regular food images, and a main effect of participant nationality [*F*_(1, 85)_ = 9.10, *p* = 0.003, $\eta_{\text{p}}^{2}$ = 0.097] with a somewhat higher overall valence score for Dutch than Thai participants. For arousal, there was a significant main effect of participant nationality [*F*_(1, 85)_ = 8.89, *p* = 0.004, $\eta_{\text{p}}^{2}$ = 0.095], indicating higher arousal scores for Dutch participants than for Thai participants. There was also an effect of food image category [*F*_(1, 85)_ = 61.33, *p* < 0.001, $\eta_{\text{p}}^{2}$ = 0.419], showing higher arousal scores for molded food images than for regular food images. There was no significant interaction between participant nationality and universal food image category [*F*_(1, 85)_ = 0.005, *p* = 0.942].

Figure 5C shows hedonic liking scores averaged for each participant nationality and universal food image category. The pattern of results was identical to that of valence ratings, with a significant interaction between participant nationality and universal food image category [*F*_(1, 85)_ = 63.78, *p* < 0.001, $\eta_{\text{p}}^{2}$ = 0.429], and main effects of both participant nationality [*F*_(1, 85)_ = 11.45, *p* = 0.001, $\eta_{\text{p}}^{2}$ = 0.119] and universal food image category [*F*_(1, 85)_ = 3152.01, *p* < 0.001, $\eta_{\text{p}}^{2}$ = 0.974].

#### Cultural Food Category

**Figure 6:**
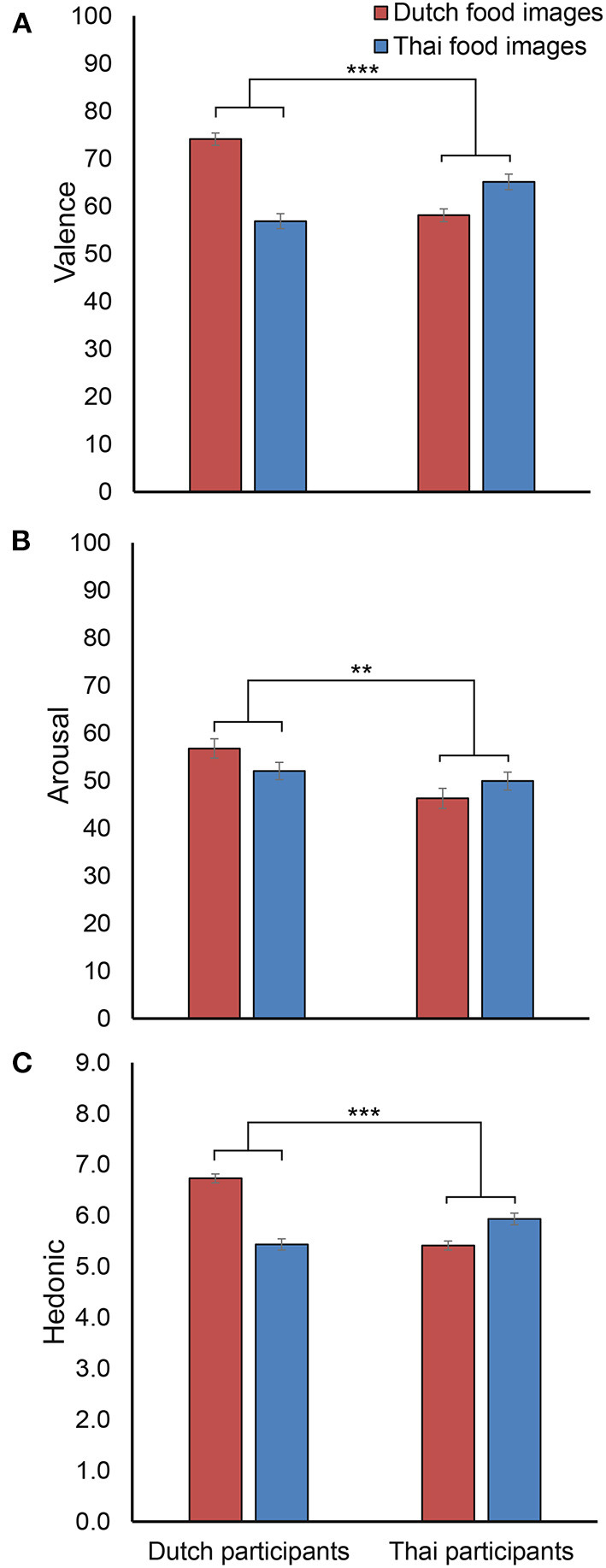
The averaged valence **(A)**, arousal **(B)**, and hedonic **(C)** scores for each participant nationality (Dutch and Thai) and cultural food image category (Dutch and Thai). Error bars indicate standard error of the mean. ^**^ and ^***^ indicate significant interactions between participant nationality and cultural food image category with *p* < 0.01 and *p* < 0.001, respectively.

The mean EmojiGrid valence and arousal scores for Dutch and Thai cultural food image categories are shown in Figures 6A,B. The two-way mixed ANOVA showed the hypothesized interaction between participant nationality and cultural food image category on valence [*F*_(1, 85)_ = 129.01, *p* < 0.001, $\eta_{\text{p}}^{2}$ = 0.603], indicating that Dutch participants rated higher valence for Dutch food images compared to Thai food images, whereas Thai participants showed the opposite pattern. Thus, participants rated food images that match their own nationality as more pleasant. There were also significant main effects of participant nationality [*F*_(1, 85)_ = 4.71, *p* = 0.033, $\eta_{\text{p}}^{2}$ = 0.053] and cultural food image category [*F*_(1, 85)_ = 22.82, *p* < 0.001, $\eta_{\text{p}}^{2}$ = 0.212] on valence scores, indicating overall higher scores for Dutch food images, and higher scores by Dutch participants. As for arousal scores, a significant interaction between participant nationality and cultural food image category [*F*_(1, 85)_ = 8.23, *p* = 0.005, $\eta_{\text{p}}^{2}$ = 0.088] indicated that Dutch participants rated relatively higher arousal for Dutch food images than for Thai food images, while Thai participants showed the opposite pattern. A significant main effect of participant nationality [*F*_(1, 85)_ = 7.06, *p* = 0.009, $\eta_{\text{p}}^{2}$ = 0.077] was found with overall higher arousal scores for Dutch participants. There was no significant main effect of cultural food image category [*F*_(1, 85)_ = 0.15, *p* = 0.704].

The mean hedonic liking scores are shown in Figure 6C. It shows the same pattern as valence scores, with a significant interaction between participant nationality and cultural food image category [*F*_(1, 85)_ = 119.71, *p* < 0.001, $\eta_{\text{p}}^{2}$ = 0.585], and significant main effects of both participant nationality [*F*_(1, 85)_ = 11.98, *p* = 0.001, $\eta_{\text{p}}^{2}$ = 0.124] and cultural food image category [*F*_(1, 85)_ = 21.75, *p* < 0.001, $\eta_{\text{p}}^{2}$ = 0.204]).

### Implicit Measures for Universal and Cultural Food Categories (Hypothesis 3 and 4)

#### Universal Food Category

**Figure 7:**
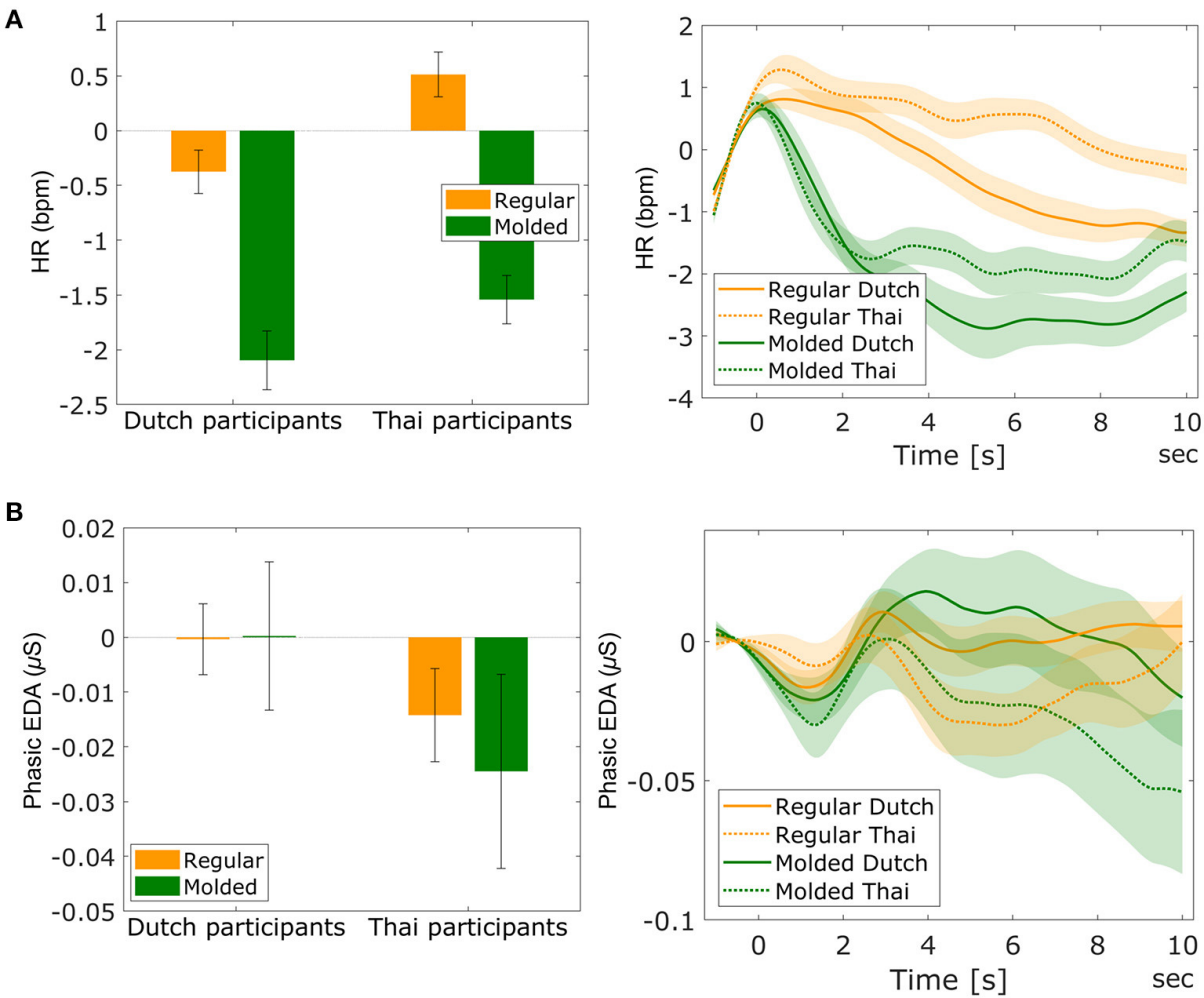
The averaged HR (**A**-left) and phasic EDA (**B**-left) for each participant nationality (Dutch and Thai) and universal food image category (regular and molded). Error bars indicate standard error of the mean. The figures at the right side show the averaged traces of HR **(A)** and phasic EDA **(B)** for each participant nationality and universal food image category during image viewing, with *t* = 0 indicating fixation cross offset, and food image onset. The light-shaded areas indicate standard error of the mean.

Figure 7A shows the averaged HR and the HR traces for each participant nationality and universal food image category. As expected, there was a significant main effect of universal food image category on HR [*F*_(1, 82)_ = 116.42, *p* < 0.001, $\eta_{\text{p}}^{2}$ = 0.587], where we observed a lower HR for molded compared to regular images. Also as expected, this effect was the same for participants of both nationalities, indicated by a lack of interaction between participant nationality and universal food image category [*F*_(1, 82)_ = 0.909, *p* = 0.343]. There was a main effect of participant nationality, [*F*_(1, 82)_ = 7.14, *p* = 0.009, $\eta_{\text{p}}^{2}$ = 0.080] with Thai participants showing higher HR than Dutch participants. Figure 7B shows the averaged phasic EDA and the phasic EDA traces for each participant nationality and each universal food image category. The two-way mixed ANOVA on phasic EDA did not show main effects of universal food image category [*F*_(1, 82)_ = 0.166, *p* = 0.684] or participant nationality [*F*_(1, 82)_ = 2.856, *p* = 0.095], and no interaction [*F*_(1, 82)_ = 0.001, *p* = 0.977].

#### Cultural Food Category

**Figure 8:**
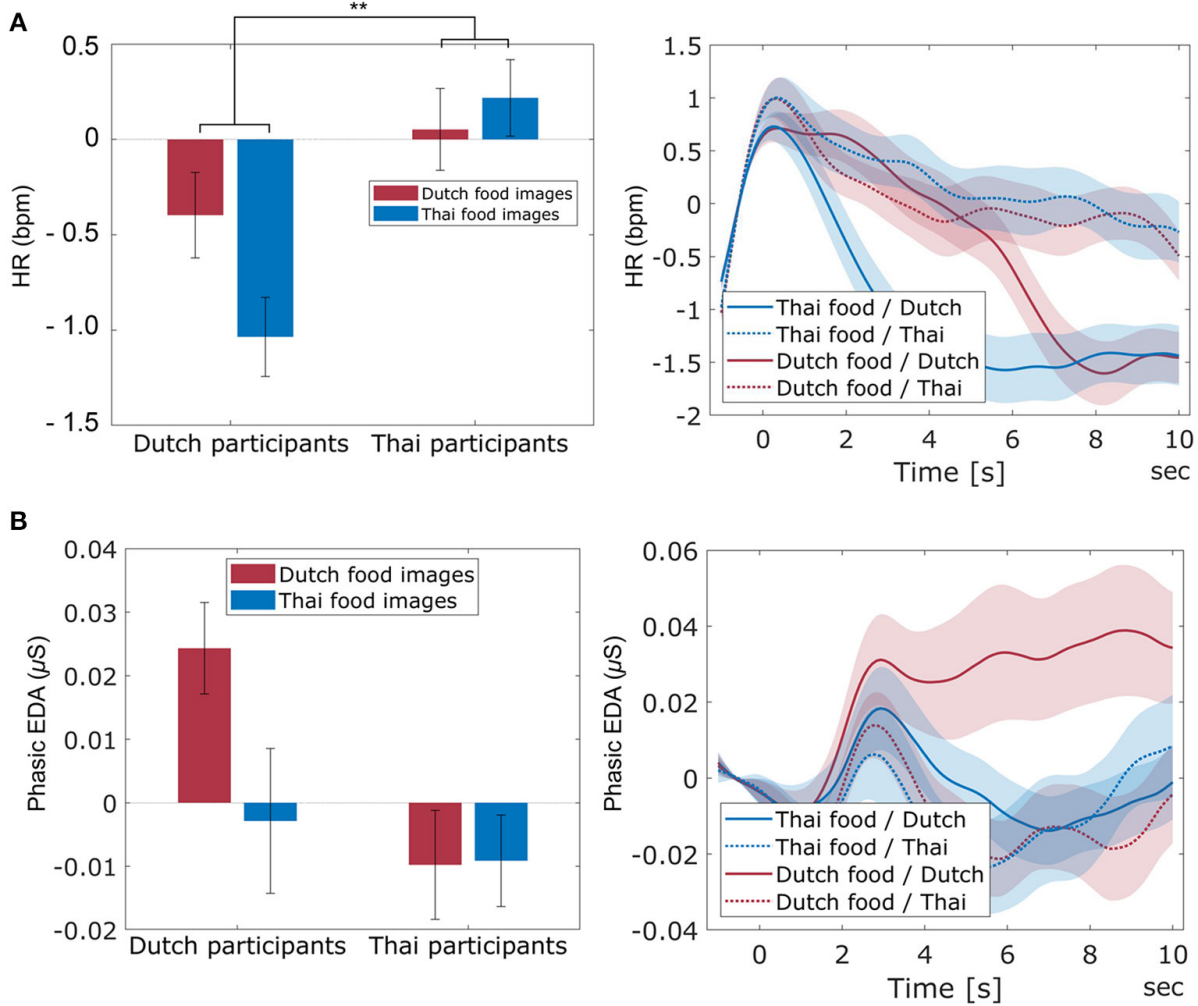
The averaged HR (**A**-left) and phasic EDA (**B**-left) across participant nationality (Dutch and Thai) and cultural food image category (Dutch and Thai). Error bars indicate standard error of the mean. The figures at the right side indicate the averaged trace of HR **(A)** and phasic EDA **(B)** across participant nationality and cultural food image category during image viewing, with *t* = 0 indicating fixation cross offset, and food image onset. The light-shaded areas indicate standard error of the mean. ^**^ indicates a significant interaction between participant nationality and cultural food image category with *p* < 0.01.

Figure 8A shows the averaged HR for each participant nationality and each cultural food image category. As expected, a significant interaction between participant nationality and cultural food image category was found on HR [*F*_(1, 82)_ = 8.50, *p* = 0.005, $\eta_{\text{p}}^{2}$ = 0.094], with Dutch participants showing lower heart rate for Thai food images compared to Dutch food images, while the pattern was opposite for Thai participants. There was no significant main effect of cultural food image category [*F*_(1, 82)_ = 2.93, *p* = 0.091], but there was a significant main effect of participant nationality [*F*_(1, 82)_ = 10.05, *p* = 0.002, $\eta_{\text{p}}^{2}$ = 0.109] with Thai participants showing higher HR than Dutch participants. Figure 8B shows the averaged phasic EDA and the phasic EDA traces for each participant nationality and each cultural food image category. For phasic EDA, no interaction between nationality and cultural food image category was found [*F*_(1, 82)_ = 3.02, *p* = 0.086], and no significant main effect of cultural food image category [*F*_(1, 82)_ = 2.74, *p* = 0.101]. There was a significant main effect of participant nationality [*F*_(1, 82)_ = 4.28, *p* = 0.042, $\eta_{\text{p}}^{2}$ = 0.050] with Dutch participants showing higher EDA than Thai participants.

### Explicit and Implicit Measures for Cultural Drinks (Hypothesis 5)

#### Explicit Measures

**Figure 9:**
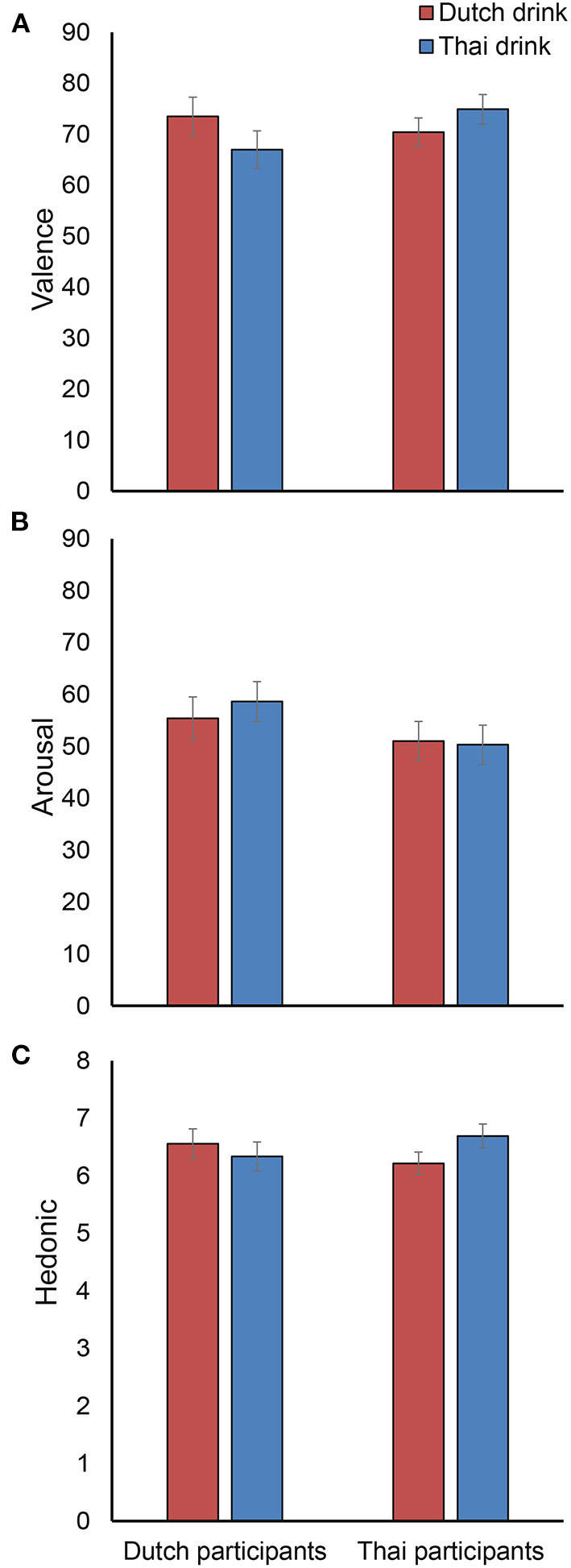
The averaged valence **(A)**, arousal **(B)**, and hedonic **(C)** scores across participant nationality (Dutch and Thai) and cultural drink category (Dutch and Thai). Error bars indicate standard error of the mean.

Figures 9A–C show respectively the average valence, arousal, and hedonic liking ratings for each participant nationality and each cultural drink. The two-way mixed ANOVAs did not show any interaction or main effects of cultural drink category and participant nationality on any of the three measures (all *p*-values are 0.094 or higher).

#### Implicit Measures

**Figure 10:**
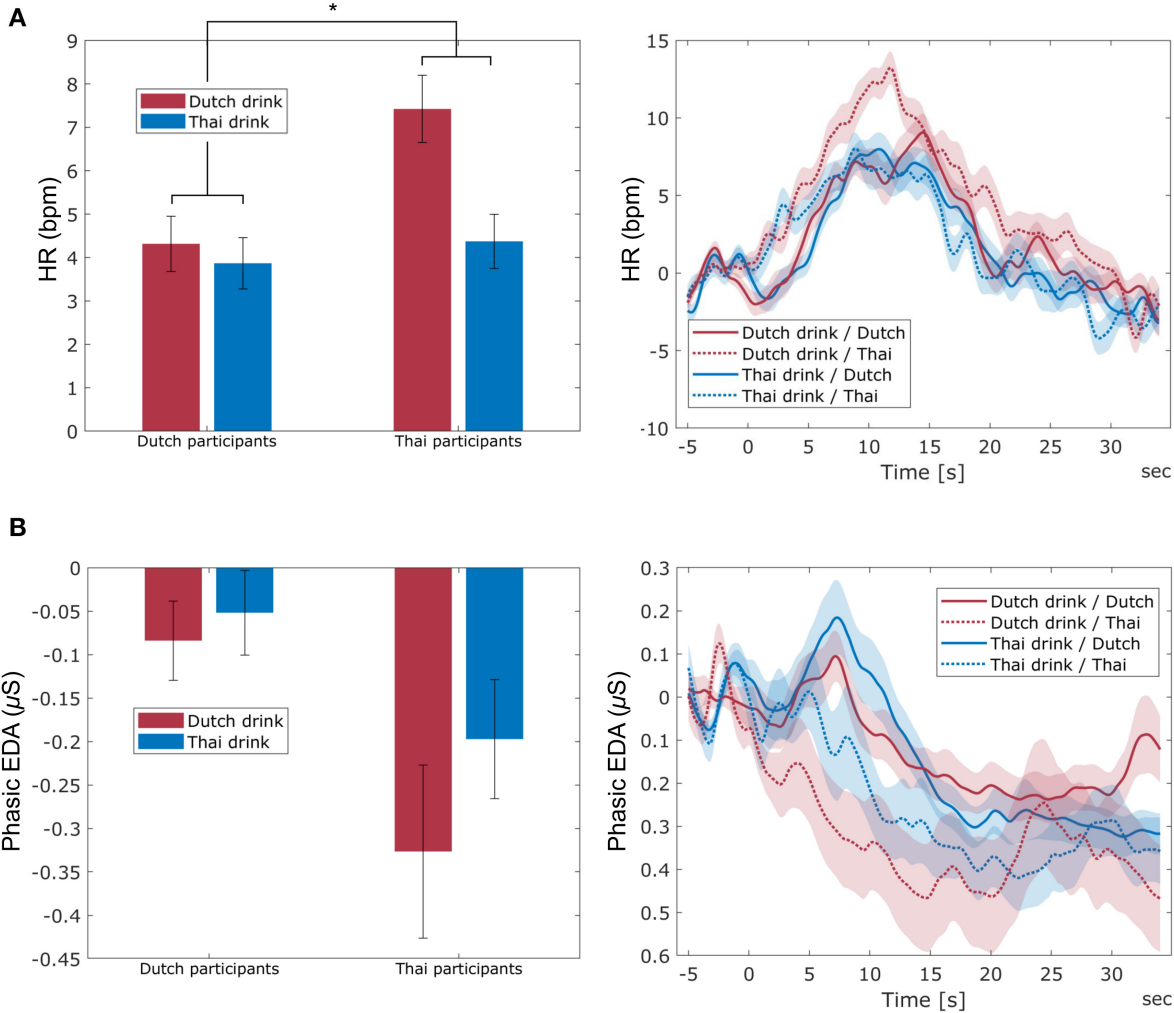
The averaged HR (**A**-left) and phasic EDA (**B**-left) across participant nationality (Dutch and Thai) and cultural drink category (Dutch and Thai). Error bars indicate standard error of the mean. The figures at the right side indicate the averaged trace of HR **(A)** and phasic EDA **(B)** across participant nationality and cultural drink category. The name of the drink is presented from *t* = −5 to *t* = 0, after which participants took one sip. The light-shaded areas indicate standard error of the mean. ^*^ indicates a significant interaction between participant nationality and cultural drink category with *p* < 0.05.

The average HR and phasic EDA for each participant nationality and each cultural drink are shown in Figures 10A,B. The two-way mixed ANOVA showed the hypothesized interaction between participant nationality and cultural drink category on HR [*F*_(1, 82)_ = 5.79, *p* = 0.018, $\eta_{\text{p}}^{2}$ = 0.066]. While both nationalities tend to show a higher HR for the Dutch drink, this is especially the case for Thai participants. Participant nationality [*F*_(1, 82)_ = 5.72, *p* = 0.019, $\eta_{\text{p}}^{2}$ = 0.065] and cultural drink category [*F*_(1, 82)_ = 10.46, *p* = 0.002, $\eta_{\text{p}}^{2}$ = 0.113] showed main effects on HR, with overall higher HR for the Dutch drink than the Thai drink, and higher HR for Thai participants than Dutch participants. For phasic EDA, there was no interaction between participant nationality and cultural drink category [*F*_(1, 82)_ = 1.07, *p* = 0.303], and no significant main effect of cultural drink category [*F*_(1, 82)_ = 2.96, *p* = 0.089]. Dutch participants showed overall higher phasic EDA than Thai participants (main effect of participant nationality: [*F*_(1, 82)_ = 5.62, *p* = 0.020, $\eta_{\text{p}}^{2}$ = 0.064].

**Figure 11:**
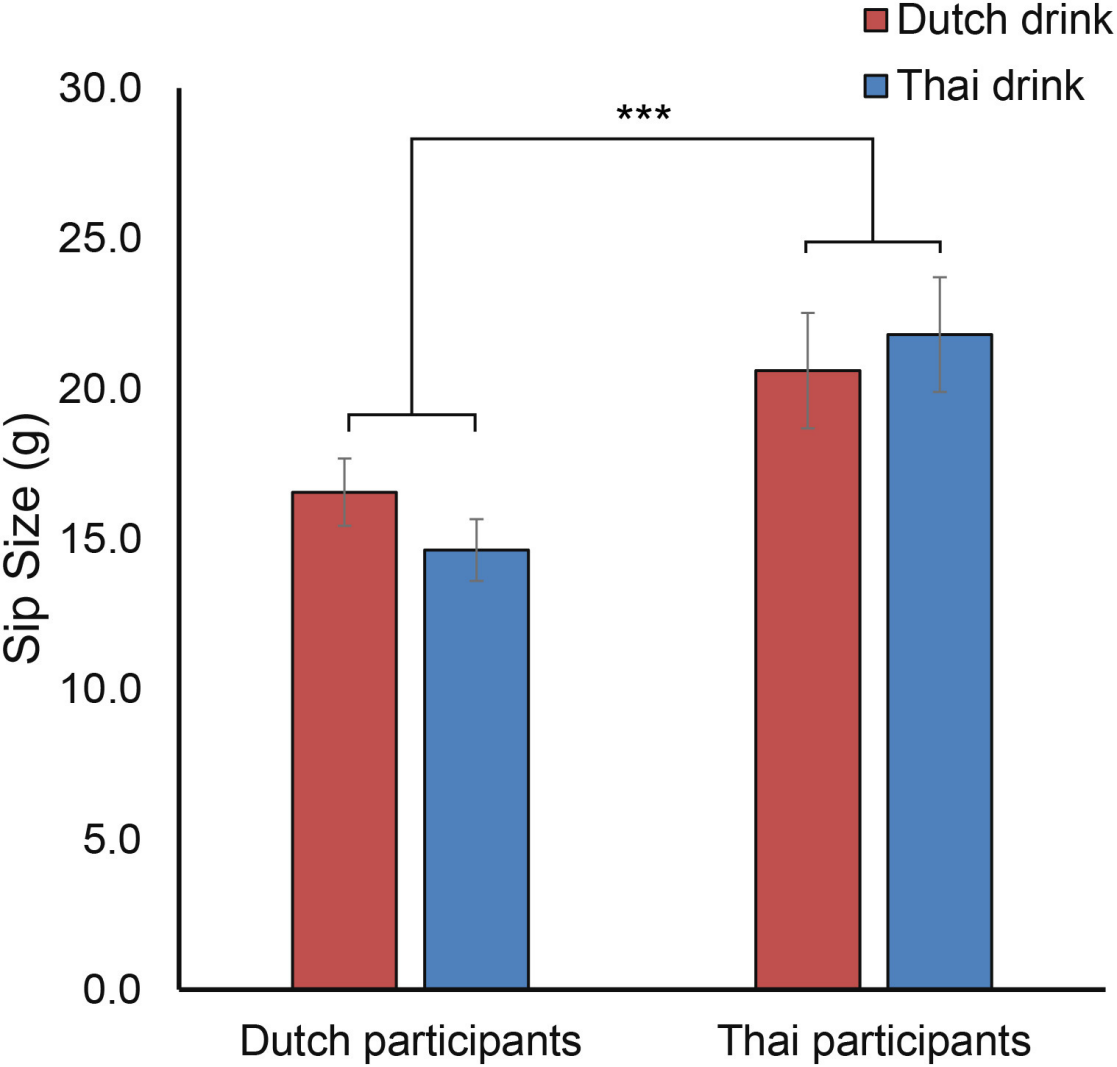
Mean sip size of Dutch and Thai drinks by Dutch and Thai participants. Error bars indicate standard error of the mean. ^***^ indicates a significant interaction between participant nationality and cultural drink category with *p* < 0.001.

Figure 11 shows the averaged behavioral measure of sip size. The expected interaction between participant nationality and cultural drink category was found [*F*_(1, 85)_ = 15.24, *p* < 0.001, $\eta_{\text{p}}^{2}$ = 0.152], indicating that Dutch participants took a larger sip of the Dutch drink than the Thai drink, while this was opposite for Thai participants. A significant main effect of participant nationality was found [*F*_(1, 85)_ = 6.97, *p* = 0.010, $\eta_{\text{p}}^{2}$ = 0.076] indicating that Thai participants took larger sips in general. There was no main effect of cultural drink category [*F*_(1, 85)_ = 0.82, *p* = 0.367].

## Discussion

The present study investigated the potential of implicit physiological measures to provide objective measures of affective food experience in contrast to explicit self-report measures, for Dutch and Thai participants. Explicit self-reports of these participants are expected to be influenced by differential cultural bias, therewith hampering comparison of food experience across cultures. Implicit physiological measures could potentially solve this problem.

Explicit self-report responses toward universal food image categories (regular and molded food images) revealed the usage of an extreme response style by Dutch participants compared to Thai participants who used a middle response style, which is consistent with the literature on response style characteristics of cultural groups (Chen et al., 1995; Johnson et al., 2005; Kaneko et al., 2019b), and confirming hypothesis 1. The valence and liking scores were higher at both ends of the scale for Dutch participants than for Thai participants. While there was no such extreme response style at both ends of the scale for arousal (where in contrast to valence, no obvious neutral location exists), Dutch participants rated universal food images more extremely on high arousal compared to Thai participants.

Familiarity ratings of the cultural food images confirmed that for Thai participants, Thai food images were more familiar than Dutch food images, while the reverse was true for Dutch participants. This confirmed that our food image stimuli were properly selected. As hypothesized in hypothesis 2, significant interactions between participant nationality and cultural food image category for self-reported valence, arousal, and hedonic liking revealed that participants rated food images from their own cultural food images as more pleasant and arousing than from the other culture. Higher liking scores for familiar foods is consistent with previous studies (Torrico et al., 2019).

As stated in hypothesis 3, HR responses were sensitive to affective food experience, as indicated by an effect of universal image type (lower HR for molded compared to regular images). The direction of this effect is consistent with literature on heart rate responses to high and low valence images (Bradley et al., 1990; Lang et al., 1993). As also stated in hypothesis 3, this effect was the same for both cultural groups, suggesting that despite the culturally-dependent difference in explicit ratings, affective food experience of universal food images (regular and molded) is the same across cultures. For cultural food images, we found the expected interaction effect between participant nationality and cultural food category on HR (hypothesis 4). Dutch participants had a lower HR in response to Thai food images compared to Dutch food images and *vice versa* for Thai participants. The direction of the effect is as expected, consistent with lower valence for unfamiliar food.

For cultural drinks, we found an interaction between participant nationality and cultural drink category on the implicit behavioral measure of sip size in the expected direction (hypothesis 5), indicating that participants of both nationalities take a larger sip of the drink from their own culture. HR also showed an interaction between participant nationality and cultural food category, where Thai participants showed a particularly strong response to the Dutch drink. These results corroborate previous results on sip size and HR (and other physiological measures) as sources of information on affective experience of taking a sip of a drink (Kaneko et al., 2019a), where sip size was smaller and HR was higher for (low valence and) high arousal drinks. In the present study, visual, olfactory and taste properties of the drinks were held constant and only the associated emotion differed. In contrast to the sip size and HR results, we unexpectedly did not find an interaction effect between participant nationality and cultural food category in explicit self-reported affective scores. Also, while familiarity scores showed the intended interaction between participant nationality and cultural food category, and Dutch participants rated the Dutch drink as much more familiar than the Thai drink, Thai participants rated the two drinks almost equal in familiarity, which is in apparent conflict with their smaller sip size and higher HR response. Note that in contrast to the implicit and explicit responses to food images, in the case of taking a sip, participants' sensory perception fundamentally changes over time. It starts with an expectation primed by the announcement of the drink name, is followed by perceiving its visual and olfactory properties, and ends with the actual taste. We speculate that the seemingly conflicting results between implicit and explicit measures are caused by their difference in time. For measures that reflect the time of taking a sip and shortly thereafter (sip size and HR), we observe a pattern in line with the hypothesis. When rating the drinks, which happens about half a minute later, Thai participants may realize they are actually familiar with the yogurt drink, and participants of both nationalities may like the taste of both drinks.

In contrast to HR, phasic EDA was not a sensitive measure of food evoked emotion in the present study, as indicated by our finding that phasic EDA responses did not differ between regular and molded food images. In addition, no interaction between participant nationality and cultural food category was found for phasic EDA; neither for images nor for drinks. While in general, there is no straightforward relation between HR and valence, studies using emotional images as stimuli consistently show valence (rather than arousal) effects, where pleasant stimuli correlate with higher heart rate acceleration than unpleasant stimuli (Hare et al., 1970; Libby Jr et al., 1973; Winton et al., 1984; Greenwald et al., 1989; Bradley et al., 1990; Lang et al., 1993, 1998; Bradley and Lang, 2000; Anttonen and Surakka, 2005; Codispoti and De Cesarei, 2007; Sokhadze, 2007). We found this HR effect as well, both in universal and cultural food categories. For EDA, consistent positive associations with arousal have been found in a range of contexts, including emotional/neutral picture viewing tasks (Greenwald et al., 1989; Lang et al., 1993, 1998) and tasting drinks (Kaneko et al., 2019a; Brouwer et al., 2020). In the present study, phasic EDA showed no (interaction with) food category effects. We did not have a clear a priori expectation that arousal should differ between cultural food image categories (familiar and unfamiliar), but we had expected higher arousal for molded food images compared to regular food images. This expectation was supported by the ratings, but the difference in rated arousal between regular and molded food images was modest compared to the difference in valence. Thus, the lack of effect in EDA may have been caused by relatively small genuine difference in arousal between our stimulus categories; they differed more in valence which, at least for images, is better captured by HR. Consistent with our findings is a study by Anderson et al. (2019). This study showed similar skin conductance levels when viewing both rotten and sweet food images, while HR was decreased when viewing molded food images compared to sweet food images.

Our analyses showed trends indicating overall higher HR and lower phasic EDA for Thai participants than for Dutch participants. Note that our HR and phasic EDA variables reflect baselined responses. We examined whether these differences in responses to food stimuli might be related to differences in overall HR and phasic EDA, which e.g., could have been caused by a difference in climate between the Netherlands and Thailand (Madaniyazi et al., 2016). However, we did not find statistically significant differences between nationalities for unbaselined HR and EDA levels.

While implicit physiological measures have been praised as objective markers of affective experience, we do not know of studies that clearly show or suggest that explicit self-reported ratings are less accurate markers of food experience than physiological measures. With our study, we attempted to fill this gap, and our results suggest that implicit physiological measures could indeed be considered as better indicators of food experience than explicit ratings. However, we should consider that we do not (cannot) know the absolute “true” emotion. Our findings could still be consistent with a genuine difference between Thai and Dutch participants with respect to the experience of viewing regular and molded food images, following the explicit ratings which are not “really” biased, but reflecting genuine differences. In this interpretation, HR would not be sensitive enough to capture this, even though HR could capture other effects that may have been stronger. Replication of our results with other types of ground truth stimuli, and relating emotions to behavior (e.g., food choice), would therefore be good to further establish the findings of the present study. For future studies, it would also be of interest to investigate if and how possible cross-cultural differences in food neophobia influence different measures of affective food experience. Finally, for adding another dimension of food experience, it would be valuable to include implicit measures of approach-avoidance motivation such as EEG alpha asymmetry (Harmon-Jones et al., 2010; Brouwer et al., 2017) or a behavioral approach-avoidance task (Solarz, 1960; Piqueras-Fiszman et al., 2014; Van Beers et al., 2020).

## Conclusion

In the present study, we examined implicit measures of food experience in a case that self-reported ratings cannot be taken at face value because of possible culturally dependent response bias. Our study confirmed the existence of such a cultural response bias and showed that only the implicit physiological measure of HR followed the prior expectations of genuine food experience. Different contexts, in this case, different types of food stimuli (images and drinks) resulted in different sensory, affective and dynamics of physiological responses. For both types of stimuli, we found the expected interaction between participant nationality and cultural food category on implicit measures (HR, and in case of tasting, sip size). This study indicates that physiological responses can be used to investigate differences in affective food experience between cultures. Especially when estimating possible acceptance of products and product promotion in cultures that strongly differ with respect to expressing affect, such as Asian and western cultures, using self-reports alone may lead to incorrect conclusions.

## Data Availability Statement

The raw data supporting the conclusions of this article will be made available by the authors, without undue reservation.

## Ethics Statement

The studies involving human participants were reviewed and approved by TNO Institutional Review Board. The patients/participants provided their written informed consent to participate in this study.

## Author Contributions

DK and AM-B: conceptualization. DK, IS, AR, and AM-B: data curation and software. DK and IS: formal analysis. DK: funding acquisition, resources, and writing—original draft. DK, IS, and AM-B: methodology, project administration, validation, and visualization. AR, AT, JvE, and AM-B: supervision and writing—review and editing. All authors contributed to the article and approved the submitted version.

## Conflict of Interest

The authors declare that the research was conducted in the absence of any commercial or financial relationships that could be construed as a potential conflict of interest.
